# The Influence of Changes in Daily Life Habits and Well-Being on Fatigue Level During COVID-19 Pandemic

**DOI:** 10.5334/pb.1259

**Published:** 2024-07-18

**Authors:** Maëlle Charonitis, Florence Requier, Camille Guillemin, Mathilde Reyt, Adrien Folville, Marie Geurten, Christine Bastin, Sylvie Willems, Vincenzo Muto, Christina Schmidt, Fabienne Collette

**Affiliations:** 1 GIGA-CRC Human Imaging, University of Liège, Liège, Belgium; 2 Psychology and Cognitive Neuroscience Research Unit, University of Liège, Liège, Belgium

**Keywords:** fatigue, mental fatigue, anxiety, lockdown, COVID-19

## Abstract

The COVID-19 pandemic and its lockdown in March 2020 have led to changes in lifestyle and increased levels of anxiety, depression, and fatigue. This survey examined a number of factors (anxiety state, sleep quality, daily activities, mental load, work-related variables) influencing mental and physical fatigue during lockdown and how these relations have evolved one year later. A cohort of 430 workers and 124 retirees were recruited in April-May 2020 (lockdown period, data set 1), and a subsample (133 workers and 40 retirees) completed the same questionnaire in April-May 2021 (data set 2). Linear regression models showed a significant subjective increase in both physical and mental fatigue in both worker and retiree groups during lockdown, and a supplementary increase in physical fatigue and anxiety level in spring 2021 compared to the lockdown period. During lockdown, anxiety level, concerns about COVID-19, work flexibility, mental load, and sleep metrics were associated with the evolution of fatigue among workers. For retirees, only anxiety and physical activity levels were linked to changes in physical fatigue. In April-May 2021, the only associations which remained significant were those in workers between fatigue and anxiety level and workload. These findings suggest that the increased fatigue levels during the lockdown are likely due to the swift and significant changes in daily routines (such as sleep patterns and work dynamics) and psychological states (including increased anxiety and concerns) prompted by the sanitary crisis. On the other hand, the increase in fatigue observed one year after the beginning of the pandemic seems to result from more psychological factors associated with the health situation.

## Impact of COVID-19 and Lockdown Measures

Due to the COVID-19 pandemic which started in China in December 2019, several countries, including Belgium, had established a mandatory lockdown to limit the spread of the virus and prevent the saturation of health care facilities (Atalan, 2020). From March 18, 2020, all residents were forbidden to go to work (except if imperative), stores were closed (to the exclusion of grocery stores), all activities were canceled, and social contacts were restricted (Service Public Fédéral Belge, s. d., 2020a, 2023). As a result of the decrease in contamination cases, a first progressive lifting of lockdown measures was put in place in Belgium on May 4th, 2020 (Service Public Fédéral Belge, 2020b). Despite this initial process, people experienced a sense of uncertainty as they continued to live in a state of anticipation, waiting for the government to enact new policies each month, often resulting in frequent backtracking (Faniel & Sägesser, 2020).

This prolonged period of social isolation and the accompanying uncertainties have significantly affected the Belgian population, resulting in anxiety and psychological distress (Glowacz & Schmits, 2020). Moreover, even without a lockdown implementation, the pandemic context itself can be seen as a mental health stressor, contributing to increased levels of uncertainty and common psychological consequences such as distress, depression, and anxiety disorders among the general population (del-Valle et al., 2022). In addition to these symptoms, increased feeling of fatigue was also frequently reported during the lockdown period (Morin et al., 2022; Torrente et al., 2022).

## Understanding Fatigue

Fatigue is a complex and multifaceted phenomenon, characterized by diverse definitions arising from its varied origins, natures, and expressions (Skau et al., 2021). Indeed, fatigue can manifest as either a pathological symptom or a transient physiological response, with its nature encompassing both physical and mental aspects, and its expressions ranging from objective measurements to subjective experiences. Commonly, fatigue is defined as a feeling of tiredness and a need for rest, highlighting the subjective nature of fatigue (Finsterer & Mahjoub, 2014). However, an objective perspective on fatigue is also recognized. Consequently, the term “fatigability” is employed to denote the measurable changes in performance resulting from fatigue, such as declines in accuracy and prolonged reaction times (Kluger et al., 2013). In addition to its expression (subjective vs. objective), fatigue is further categorized into two distinct natures: (1) mental/cognitive fatigue, characterized by a decrease in attention and motivation, independent of any cognitive deficit, and (2) physical fatigue, which is based on a lack of energy at the muscular level (Chaudhuri & Behan, 2000; Dobryakova et al., 2013).

Concerning its origin, fatigue can be manifested as normal transient physiological response following a prolonged activity or as a persistent pathological symptom. This latter aspect can stem to a physical disease (i.e., it is one of the listed symptoms of COVID-19; Sharma et al., 2022; Wensink et al., 2023), a manifestation of an underlying psychological condition (such as depression; Baldwin & Papakostas, 2006; Demyttenaere et al., 2005), or a combination of both simultaneously. Typically, the manifestation of fatigue co-occurs with a spectrum of overlapping symptoms, including stress, anxiety, depression, sleep disorders, and pain. Indeed, anxiety is known to have considerable effects on both physical and mental health (Juster et al., 2010). It depletes mental resources, impairs attention and executive functions, and triggers the body’s prolonged stress response, all of which can increase fatigue levels (Eysenck & Derakshan, 2011; Moran, 2016). Similarly, poor sleep quality has a substantial impact on fatigue, by reducing both mental and physical energy (Åkerstedt et al., 2004; Boolani & Manierre, 2019). During the lockdown, disruptions in daily routines and increased stress levels may have negatively impacted sleep patterns, exacerbating feelings of fatigue. But fatigue is also known to be influenced by various other factors including age, gender, physical condition, sleep quality, diet, personality traits, and general health status (Finsterer & Mahjoub, 2014). For example, fatigue can be influenced by gender through hormonal fluctuations, such as those occurring throughout the menstrual cycle (Li et al., 2020; Pallavi, 2017). Another factor that can contribute to fatigue is the individual’s overall health status, particularly the presence of inflammation (De Raaf et al., 2013). Inflammation triggers the release of cytokines, which can disrupt normal physiological processes and contribute to feelings of fatigue, especially in chronic inflammatory conditions such as autoimmune diseases or infections (Roerink et al., 2017). However, it’s important to acknowledge that these examples represent only a fraction of the complex interplay of factors contributing to fatigue, and further research is needed to unravel the full spectrum of mechanisms involved. Understanding the multifaceted nature of fatigue requires a comprehensive exploration of its interplay with physiological, psychological, and environmental variables. Consequently, it seems reasonable to assume that the overall pandemic context and the associated changes in daily activities and well-being may be partially responsible for increased fatigue symptoms in the population. For example, a study of Field et al. (2021) showed that psychological variables explaining fatigue during lockdown were depression (37%), sleep disturbance (12%), and anxiety (1%), while health activities (exercise, physical contact, self and spiritual care) explained 11% of the variance on fatigue scores.

The literature of mental fatigue suggests that undertaking cognitively demanding tasks amidst the pandemic-related adjustments, such as remote work, face covering and social distancing measures, could lead to heightened mental fatigue (van der Linden et al., 2003; Boksem et al., 2005, 2006). Consequently, these changes in daily habits may contribute, along with high anxiety and depression levels, to a sense of decreased attentional and executive functioning efficiency, further exacerbating the experience of fatigue (Fiorenzato et al., 2021).

Additionally, some studies have shown that a sedentary lifestyle is associated with increased risk of experiencing feelings of physical fatigue (Puetz, 2006). Thus, it seems plausible that increased physical fatigue during lockdown may be related to the lack of physical stimulation resulting from staying at home. Indeed, some authors found that the pandemic has tended to disturb physical activity of the individuals by significantly decreasing its level (Ammar et al., 2020). However, other studies have identified an increase in physical activity practice (Gallè et al., 2020). This mix result can be attributed to a dynamic pattern, initially marked by a decrease in physical activity due to the lockdown onset. However, as individuals gradually adapted themselves to the new sanitary situation, a subsequent rise in physical activity emerged, with people engaging in home workouts or opting for outdoor activities like running or walking (López-Bueno et al., 2020). Unfortunately, physical fatigue in the context of the COVID-19 pandemic remains largely unexplored. Specifically, the existing literature lacks clarity on the precise causes of emergence of fatigue and the factors contributing to its persistence.

## Aim and Hypotheses

The pandemic context may serve as a natural laboratory to examine how adapting to rapid changes in lifestyle and daily life habits impacts the perceived fatigue state. Therefore, the aim of this study is to investigate potential risk factors (including demographics, anxiety state, daily activities, mental load, sleep metrics, and work investment) for increased fatigue level during and after the lockdown period among both workers and retirees. As previously mentioned, changes in daily routines such as sleep pattern may have intensified feelings of fatigue.

As a main research hypothesis, we anticipate potential associations between increased fatigue level and risk factors specific to the lockdown (i.e., sudden change in life habits and worries about health situation). However, how fatigue levels and its relationship with risk factors will evolve one-year post lockdown remains elusive and we consider this research question as more exploratory, without any firm predictions on the relationships between fatigue level and risks factors. Indeed, with the end of social isolation and the development of coping strategies to manage the health situation, we may posit a decrease in fatigue levels and no more association with risk factors specific to lockdown. Alternatively, if stressors related to the health situation (such as fear of the virus transmission or other distressing conditions associated with the pandemic) serve as primary drivers of increased fatigue, fatigue might remain high at the one-year follow-up, and its associations with daily life aspects (or at least some of them) will likely remain significant. Finally, we acknowledge the possibility of sustained high levels of fatigue at both time points, while the underlying factors explaining fatigue may differ. For instance, fatigue might be primarily associated with the lockdown measures in 2020, whereas one year later, it may be predominantly attributed to the prolonged duration of the pandemic and ongoing impact of the health situation.

Additionally, we hypothesize that the population would react differently to the pandemic depending on their working status and their unique associated challenges, resulting in distinct impacts on their levels of fatigue. Indeed, workers have been previously associated with increased fatigue due to work characteristics such as work stress (Bültmann et al., 2002) or job demands (De Lange et al., 2009). Therefore, workers are likely to experience heightened fatigue due to abrupt shift to remote work. In contrast, retirees face different challenges that may influence their fatigue levels. The transition from an active work life to retirement often involves changes in daily routines and potential reductions in physical activity (S. Chung et al., 2009). Social isolation during the pandemic could have intensified feelings of anxiety and uncertainty about health risks (Parlapani et al., 2020), particularly as older adults are more vulnerable to COVID-19 complications. These factors may lead to increased fatigue, but through different mechanisms compared to those affecting workers. Studying these two groups separately is essential because the underlying causes and manifestations of fatigue are likely to differ. For workers, fatigue may be more closely related to occupational stressors and the disruption of work habits, while for retirees, it may be more associated with changes in lifestyle, physical activity levels, and social interactions. Furthermore, individuals of different ages typically experience varying levels of fatigue even under normal conditions (De Jong et al., 2018; Galland-Decker et al., 2019; Gilsoul et al., 2021). By analyzing these groups independently, we can gain a clearer understanding of the specific factors contributing to fatigue in each population.

## Method

### Participants

Participants were recruited via social networks and email contacts from our pre-existing volunteer database. The study was conducted after approval by the Ethics Committee of the Faculty of Psychology, Speech Therapy and Educational Science of Liège University. All participants gave their electronic informed consent following information about the objectives of the study. The informed consent process involved providing detailed information about the study’s objectives and subsequent communications for research purposes, including potential follow-up surveys or inquiries. No financial compensation was provided for participation to the study.

Participants were asked to complete an online questionnaire on two occasions: between April 9^th^ and May 19^th^, 2020 (data set 1) and one year later between April 2^nd^ and May 22^nd^, 2021 (data set 2). The period for the first fill-in of the questionnaire was characterised by full-lockdown conditions and also by culminating diagnosed cases and death rates identified as the first peak of COVID-19 infections in Belgium (Molenberghs et al., 2022). The period of the second fill-in took place in a period without lockdown and the reopening of business but with many strict sanitary rules including mandatory teleworking and forbidden outdoor gatherings exceeding 10 people.

**Figure 1:**
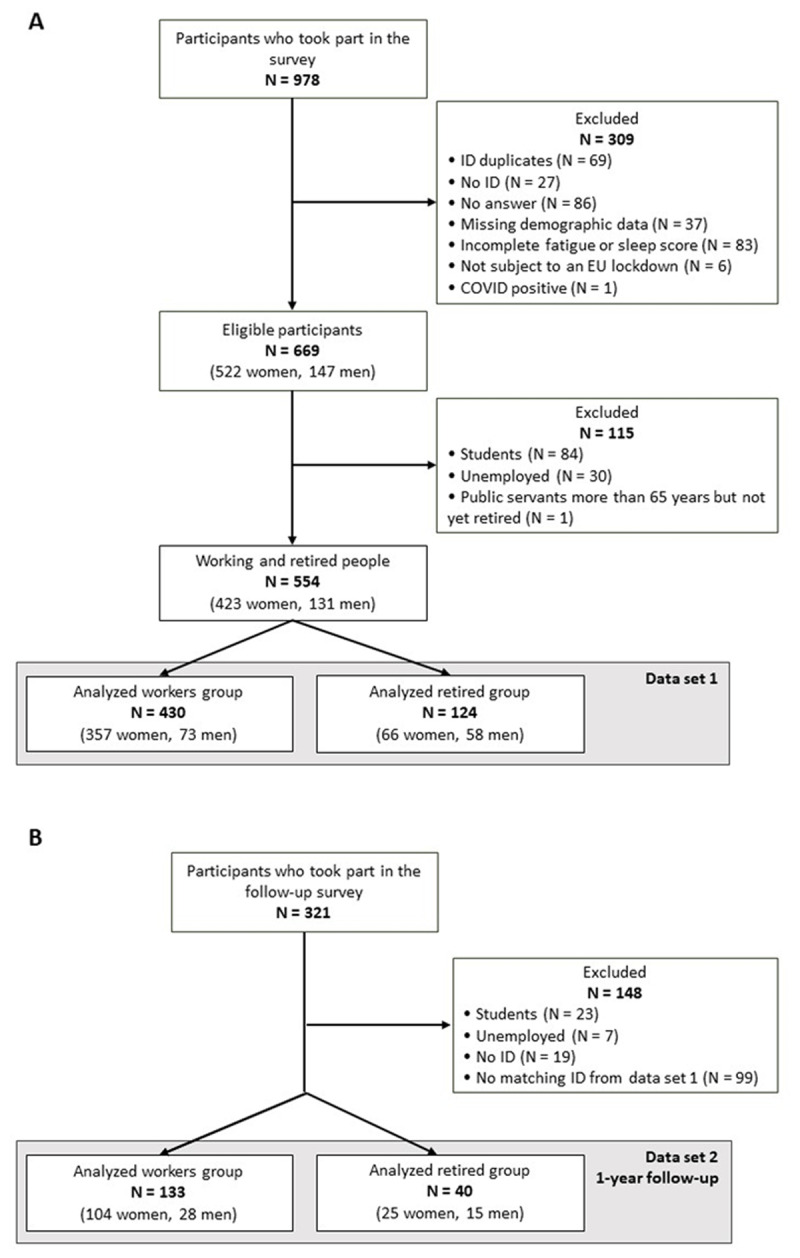
Flowchart of Participants for **(a)** data set 1 and **(b)** data set 2.

For data set 1, 978 people initially took part in the survey (see Figure 1 for the Flowchart). After ID duplicates removal (N = 69), 233 subjects were excluded because of mandatory data missing (i.e., no ID, no demographic data, none/incomplete fatigue scores…). We have also removed participants with a previous diagnosis of COVID-19 confirmed by polymerase chain reaction test (PCR) (N = 1) and participants who were not subject to a lockdown while completing the questionnaire in 2020 (N = 6). Students (N = 84), unemployed (N = 30) and public servants of retirement age who are still working (N = 1) were also removed from the sample. In that way, the cohort was composed exclusively of active workers and retirees. As active workers and retirees represent distinct life stages with significant differences in daily routines, responsibilities, and social interactions, the analyzes were done on each group separately. Moreover, even under normal circumstances, individuals of different ages exhibit varying levels of fatigue (De Jong et al., 2018; Galland-Decker et al., 2019; Gilsoul et al., 2021). Considering these baseline differences in fatigue levels across age groups, we anticipate similar age-related specificities to the pandemic-related stressors.

The final sample for data set 1 (see Figure 1A) consisted of 554 participants aged between 20 and 87 years old, that were subdivided in two groups: workers (N = 430; mean age = 40.4, SD = 12.2) and retirees (N = 124; mean age = 68.9, SD = 6.1). Participants were French speakers living in Belgium (87.73%), except for a few participants established in different European countries also subject to a lockdown when answering the questionnaire (France N = 63 (11.37%), Switzerland N = 2 (0.36%), Germany N = 1 (0.18%), Luxembourg N = 1 (0.18%) and Spain N = 1 (0.18%)). Demographic characteristics for the two groups are detailed in Table 1.

**Table 1:** Demographic information for workers **(a)** and retirees **(b)**: Data set 1.


GROUP CHARACTERISTICS	MODALITIES	N (%)

**(a) Workers (N = 430)**		

**Age**, *years*	40.43 (SD = 12.16) (range 20–69 years)	

**Gender**	Male	73 (16.98)

	Female	357 (83.02)

**Educational level**	Elementary school	1 (0.24)

	High school	24 (5.9)

	Undergraduate degree (shortype)	105 (24.88)

	Postgraduate degree (longtype)	292 (69.19)

**Teleworking**, *yes*		316 (73.49)

**(b) Retirees (N = 124)**		

**Age**, *years*	68.86 (SD = 6.13) (range 52–87 years)	

**Gender**	Male	58 (46.77)

	Female	66 (53.23)

**Educational level**	Elementary school	3 (2.42)

	High school	26 (20.97)

	Undergraduate degree (shortype)	48 (38.71)

	Postgraduate degree (longtype)	44 (35.48)


All individuals who completed the questionnaire in 2020 were invited to take part in a follow-up survey in April-May 2021. Out of the initial 321 participants (see Figure 1B), 10 were excluded due to missing identification, 99 due to ID mismatches from data set 1. Additionally, students (N = 23) and unemployed participants (N = 7) were removed from the data set, mirroring the criteria applied to data set 1. Ultimately, 133 workers (age range: 24–67 years; mean age = 44.3, SD = 11.2) and 40 retirees (age range: 56–83 years; mean age = 68.2, SD = 5.6) fully answered the second survey. Among these participants, 21 were from France (12.14%), and one from Switzerland (<.01%). These participants constitute data set 2 (see Table 2 for demographic characteristics of data set 2).

**Table 2:** Demographic information for workers **(a)** and retirees **(b)**: Data set 2.


GROUP CHARACTERISTICS	MODALITIES	N (%)

**(a) Workers (N = 133)**		

**Age**, *years*	44.32 (SD = 11.23) (range 24–67 years)	

**Gender**	Male	28 (21.05)

	Female	104 (78.20)

**Educational level**	Elementary school	0

	High school	6 (4.55)

	Undergraduate degree (short)	34 (25.76)

	Postgraduate degree (long)	92 (69.17)

**Teleworking**, *yes*		87 (65.41)

	

**(b) Retirees (N = 40)**		

**Age**, *years*	68.20 (SD = 5.64) (range 56–83 years)	

**Gender**	Male	25 (62.50)

	Female	15 (37.50)

**Educational level**	Elementary school	2 (5.00)

	High school	6 (15.00)

	Undergraduate degree (short)	18 (45.00)

	Postgraduate degree (long)	14 (35.00)


While the majority of our participants were in Belgium during the lockdown, approximately 12% were located in other European countries where comparable lockdown measures were put in place (Conseil Fédéral Suisse, 2020; Goberno de España, 2020; Gouvernement du Grand-Duché du Luxembourg, 2020; Macron, 2020; Schrack et al., 2020). Consequently, we opted to follow the rationale of our previous studies (Cellini et al., 2021; Folville et al., 2023) by including these participants in the analyses (after verifying the absence of outliers among participants outside Belgium). We considered that any minor variations in the strictness of lockdown measures across countries would likely have minimal impact on the parameters studied. Similarly, although the vaccination campaign in Belgium started concurrently with the completion of our follow-up survey, its impact on our assessments should be limited. Indeed, the campaign primarily targeted healthcare personnel, representing only a small fraction of our participant sample (Sciensano, 2021).

### Material and procedure

The questionnaire was administered as an online survey, designed, and implemented through the GDPR compliant system integrated into the intranet of the Faculty of Psychology, Speech Therapy, and Educational Science at the University of Liège. The survey was made available online from April to May 2020 and from April to May 2021. For data set 1, workers and retirees were solicited to complete the same questionnaire twice, considering their condition before the lockdown onset (i.e., “before” period, defined as the period between March 1^st^, 2019, and March 1^st^, 2020) and during the first lockdown restrictions (i.e., “during” period, defined as from March 13^th^, 2020, to time of survey completion). For data set 2, participants were contacted one year later to respond to questions pertaining to the current period (i.e., “after” period, defined as the period beginning in April 2021). This latter period is characterized by the end of the lockdown, but with the persistence of uncertainties and sanitary measures due to the health situation. The objective was to highlight which factors related to the new situation are associated with the development and evolution of mental and physical fatigue, separately in the two populations.

The survey administered in 2020 addressed other research questions than the one discussed here (see the “Transparency and Openness” section). From the whole questionnaire, we will consider the following categories for the current study: demographics, anxiety state (only assessed for the periods “during” and “after” lockdown), daily activities, investment at work, sleep characteristics, subjective mental load, and subjective fatigue feeling (assessed three times: “before”, “during”, and “after” lockdown). Fatigue (either physical or mental) is considered as the main dependent variable, that might be modulated by demographics and/or lockdown/post lockdown related changes in other independent variables. The specific variables associated with each category used in statistical analyses are detailed below and summarized in Table 3. Examples of items for each questionnaire can be found in **Appendix A**.

**Table 3:** Variables used in statistical analyses for data sets 1 and 2.


CATEGORIES	VARIABLES	INSTRUMENTS

**Demographics**	Age	–

	Gender	–

	Education	–

	Working status^1^	–

**Anxiety state**	General anxiety	State–Trait Anxiety Inventory (STAI)^2^

	COVID–related anxiety	Visual analog scale (0 to 100)

***Work–related activities***	*Home office*	Yes/No question

	*Effort*	Visual analog scale (0 to 100)

	*Flexibility*	Visual analog scale (0 to 100)

	*Work activity*	Visual analog scale (0 to 100)

**Leisure–related activities**	Screen exposure	Estimated daily duration (in minutes)

	Outdoor	Estimated daily duration (in minutes)

	Sport	Estimated daily duration (in minutes)

**Mental load**	House duties	Visual analog scale (0 to 100)

	Social interactions	Visual analog scale (0 to 100)

	Work/volunteering	Visual analog scale (0 to 100)

	Self–centered leisure activities	Visual analog scale (0 to 100)

**Sleep and sleepiness**	Sleepiness	Epworth Sleepiness Scale (ESS)^3^

	Sleep quality	Pittsburgh Sleep Quality Index (PSQI)^4^

	Naps	Calculation based on naps quantity and duration^5^

**Fatigue**	Physical	Multidimensional Fatigue Inventory (MFI)^6^

	Mental	Multidimensional Fatigue Inventory (MFI)^6^


*Note*. In italic, workers only. ^1^Working status was used to include workers and retirees in separate analyses. ^2^Anxiety level was measured by the 6-item short form of the STAI (Spielberger, 1983; Marteau & Bekker, 1992); ^3^Measured by ESS (Johns, 1991); ^4^Sleep related scores were extracted from PSQI (Buysse et al., 1989); ^5^Nap Score calculation based on quantity and duration (see Appendix B for detailed presentation); ^6^Fatigue was measured by MFI (Smets et al., 1995), which provides separate scores for physical and mental fatigue.

#### Predictors

**Demographics**. Sociodemographic characteristics concerned age (in years), gender (female, male) and working status (worker, retiree). Educational level was determined by asking the highest completed level of education according to the Belgian classification system: namely, elementary school (1), high school (2), undergraduate degree (3), and postgraduate degree (4).

**Anxiety state**. General anxiety state was evaluated using the 6-item short form of the State-Trait Anxiety Inventory (STAI) (Marteau & Bekker, 1992) (Cronbach’s α: 0.87 for workers, 0.83 for retirees). Participants had to rate each item on a 4-point scale ranging from 1 (not at all) to 4 (very much), with high scores reflecting high anxiety levels. COVID-related anxiety was assessed with ratings on a visual analog scale ranging from 0 to 100 (100 = the highest fear).

**Work-related activities**. Participants were asked to report work characteristics: teleworking from March 2020 (yes/no; the data is not available for the pre-lockdown period), level of effort needed to do the job, work schedule flexibility, and how professionally busy at work (% of the workday spent actually working). These last three variables were assessed using visual analog scales (0 to 100, for example, 0 “no effort at all” to 100 “a lot of effort”). This section was not administered to retired participants.

**Leisure-related activities**. Questions covered sports, outdoor activities, and use of screen devices. The measures were expressed as the estimated daily duration of each activity (in minutes).

**Mental load**. Mental load was assessed for the situations of house duties, social interaction, work (for workers only), and self-centered leisure activities. Participants had to report their level of perceived mental load in the last few days for each situation with visual analog scales ranging from 0 “high mental load” to 100 “low mental load”.

**Subjective sleep quality and sleepiness**. The Pittsburgh Sleep Quality Index (PSQI) (Buysse et al., 1989) was used to measure specific sleep-related components, namely: subjective sleep quality (C1), sleep latency (C2), sleep duration (C3), sleep efficiency (C4), sleep disturbance (C5), the use of sleep medication (C6), and daytime dysfunction (C7). Each component ranges from 0 to 3, with a score of 0 indicating no sleep difficulty and 3 severe difficulties. The Epworth Sleepiness Scale (ESS) (Johns, 1991) was administered to assess the average rest propensity in different daily situations. Total score ranges from 0 to 24, with a higher propensity of daytime sleepiness associated with higher scores (Cronbach’s α: 0.77 for workers, 0.76 for retirees).

Finally, participants were asked to report their napping habits. A nap score was calculated based on the quantity of naps per week (ranging from 1 “less than once a week” to 7 “more than once a day”) and their duration (ranging from 0 “less than 10 minutes” to 1 “more than 2 hours”). Total nap score is the sum of the scores obtained for quantity and duration, ranging from 1 to 8 (see **Appendix B** for a detailed presentation of the nap score).

#### Outcomes

**Subjective physical and mental fatigue**. Fatigue was assessed using the Multidimensional Fatigue Inventory (MFI) (Smets et al., 1995). The MFI is a 20-item questionnaire that encompasses five dimensions related to fatigue state (4 items in each): general fatigue, physical fatigue, mental fatigue, reduced activities, and reduced motivation. For every statement, participants had to select the appropriate number according to the scale: 1 (strongly disagree) to 5 (strongly agree). In order to characterize the evolution of fatigue due to the COVID-19 lockdown as well as one year later, all participants had to complete the scale three times, corresponding to their state before, during, and after the lockdown period. The dimensions of physical and mental fatigue (scores ranging from 4 to 20) will be used here (Cronbach’s α for physical fatigue: 0.81 for workers, 0.79 for retirees; for mental fatigue: 0.85 for workers and 0.78 for retirees).

### Analyses

#### Missing data management

Concerning the management of missing data, in the case of item-missing level, a total/average score was calculated for all subjects from the moment the subject had completed an item. If the subject did not answer all the items, the final score is the reflection of the items they answered (Bernaards & Sijtsma, 2000). More precisely: (i) if the final score considered is an average, the average corresponds to the average of the items answered, (ii) if the final score considered is a sum, then the average is used first and then multiplied by the number of items in the questionnaire. In the case of concept-missing level, a pairwise deletion (multiple imputation) method was used to deal with missing data (Newman, 2014). This management of missing data does not apply to demographic and fatigue variables. Participants with missing data for these scores were explicitly excluded from the study, as previously mentioned in the participants’ section.

#### Statistical Analyses

Data were analyzed using JASP 0.16.2 (Windows) and MATLAB R2017a (9.2). All statistics have been computed on workers and retirees separately. Our analyses will primarily focus on the time periods pre and during COVID-19 (data set 1), as well as during and post COVID-19 (data set 2). This decision is to ensure a more focused investigation into the specific periods directly influenced by the pandemic, aligning with our research objectives centered on understanding the immediate changes during the lockdown and the subsequent phase. However, exploratory mixed effect model analyses can be found in **Appendix C** in order to analyze the evolution of both physical and mental fatigue across three time-points (before, during, and post-lockdown) for both workers and retirees.

In data set 1, paired sample or Wilcoxon’s t-tests (depending on normality) were used to compare scores reported for fatigue feeling, daily activities, sleep and sleepiness, work investment, and mental load in the periods “before” and “during” the lockdown. When a significant difference was observed (*p* < .05), delta scores were created by subtracting the values pre-lockdown from those associated with lockdown period. The delta scores of these variables were next used in the linear regression models. By including only variables impacted by the lockdown and utilizing delta scores, we were able to significantly reduce the volume of statistical tests conducted, and to assess our hypothesis of a relationship between changes induced by the COVID-19 situation and changes in fatigue levels.

To determine risk factors for fatigue, we ran six linear regression models with delta scores for physical and mental fatigue separately set as dependent variable. The raw scores for demographic, anxiety state and teleworking variables, and the delta scores for the domains of leisure and work-related activities, sleep characteristics and mental load have been introduced as covariates or factors (depending on their continuous or categorical nature) in separate models using the simultaneous (forced entry) method, in which all predictor variables are entered simultaneously in a single step into the regression model regardless of their statistical significance.

Linear regression models allowed us to evaluate the contribution of each component within a domain that is associated with a change during the lockdown (e.g., general anxiety is a component of the anxiety state domain). Adjusted R-square was added to specify the explained variance of each model. The model summaries of the linear regression models are presented in **Tables D1** (for data set 1) and **D2** (for data set 2) within **Appendix D**. While complete models are reported for thoroughness, the focus of interest lies specifically on the variables within the categories, rather than solely on the categories themselves.

The same procedure was applied for data set 2 to compare scores between the lockdown period and one year later; and delta scores were created when a significant difference was observed, including for anxiety state for which this time delta scores were also computed for affective variables as two measures were available (“during” and “after” the lockdown period). Given the substantial disparity in sample size between data set 2 and data set 1, the analyses resulting from data set 2 will be considered exploratory.

### Transparency and openness

Data and analysis code are openly available on OSF (https://osf.io/4ef2s/?view_only=1ecf3adac21944e68612a0b07dbe363b). Design and analyses of the present study were not preregistered. The survey administered in 2020 comprised several sections and addressed other research questions than the one discussed here (see **Appendix E** for a detailed presentation). Two publications have already emerged from data set 1, one related to sleep characteristics during lockdown (Cellini et al., 2021) and the other examining memory for autobiographical events during the same period (Folville et al., 2023).

## Results

### Data set 1. Score comparison: before and during the lockdown

In the first step, we compared scores reported before and during lockdown in 2020 to determine variables affected by the health situation and lockdown that will be used in the regression analyses (see Tables 4 and 5, for workers and retirees respectively). As it is difficult to precisely assess anxiety state retrospectively, no changes in anxiety levels can be computed.

**Table 4:** Raw values and statistical outcomes of the paired sample t-tests/Wilcoxon tests (depending on the normality) for **workers**.


	BEFORE LOCKDOWN (INITIAL SAMPLE, N = 430)	DURING LOCKDOWN (INITIAL SAMPLE, N = 430)	BEFORE VS DURING (INITIAL SAMPLE, N = 430)	DURING LOCKDOWN (FOLLOW-UP SAMPLE, N = 133)	1Y POST LOCKDOWN (FOLLOW-UP SAMPLE, N = 133)	DURING VS POST (FOLLOW-UP SAMPLE, N = 133)

	MEAN (SD) MIN-MAX	MEAN (SD)MIN-MAX		MEAN (SD) MIN-MAX	MEAN (SD) MIN-MAX	

**FATIGUE**						

Physical fatigue	9.68 (3.51) 4.00–20.00	10.72 (3.76) 4.00–20.00	**W = 22460.50**, **p < .001**	10.02 (3.53) 4.00–20.00	11.66 (4.10) 4.00–20.00	**W = 2050.50, p < .001**

Mental Fatigue	9.34 (3.36) 4.00–19.00	11.37 (4.13) 4.00–20.00	**W = 15936.00**, **p < .001**	10.82 (3.98) 4.00–20.00	10.58 (4.11) 4.00–20.00	t = 0.68, p = .50

**ANXIETY STATE**						

General anxiety	–	13.07 (4.23) 6.00–24.00	–	12.11 (3.80) 6.00–23.00	13.50 (2.72) 9.00–20.00	**t = –3.97**, **p < .001**

COVID-related anxiety	–	58.04 (29.01) 0–100	–	55.86 (30.62) 0–100	40.42 (29.82) 0–100	**t = 4.83**, **p < .001**

**WORK ACTIVITIES**						

Work from home, yes (%)	–	73.49	–	77.44	65.41	**χ^2^ = 35.31**, **p < .001**

Effort	62.02 (23.44) 0–100	61.90 (31.50) 0–100	W = 33310.00, p = .15	60.83 (32.88) 1–100	64.76 (25.87) 2–100	t = –1.12, p = .27

Flexibility	51.01 (34.26) 0–100	72.27 (32.30) 0–100	**W = 9802.50**, **p < .001**	74.98 (32.75) 0–100	57.44 (35.74) 0–100	**W = 5016.00, p < .001**

Activity	84.47 (46.90) 0–100	58.75 (33.89) 0–100	**W = 55115.00**, **p < .001**	60.63 (34.38) 0–100	81.89 (23.34) 0–100	**W = 1165.00, p < .001**

**LEISURE ACTIVITIES**						

Screen exposure, *min-24h*	305.71 (216.14) 1–960	357.87 (224.10) 0–960	**W = 13260.50**, **p < .001**	377.66 (217.40) 1–960	371.25 (229.79) 2–840	W = 3916.50, p = .919

Outdoor activities, *min-24h*	65.16 (65.65) 0–480	79.85 (84.32) 0–540	**W = 30817.00**, **p = .012**	78.05 (79.66) 0–360	50.58 (46.12) 1–300	**W = 4249.00, p = .03**

Sports, *min-24h*	38.85 (51.81) 0–480	37.27 (41.74) 0–360	W = 24705.50, p = .76	39.48 (41.59) 0–210	33.91 (51.71) 0–400	W = 4102.50, p = .08

**MENTAL LOAD**						

House duties	50.83 (28.21) 0–100	63.96 (29.53) 0–100	**W = 24802.00**, **p < .001**	68.21 (28.36) 0–100	46.96 (29.79) 0–100	**W = 6401.00**, **p < .001**

Social interactions	75.70 (23.57) 0–100	69.24 (28.63) 0–100	**W = 38719.50**, **p = .001**	71.36 (29.37) 0–100	69.90 (27.61) 0–100	t = .59, p = .56

At work	80.21 (19.35) 4–100	55.63 (31.71) 0–100	**W = 62791.50**, **p < .001**	63.35 (26.24) 0–100	61.23 (31.71) 0–100	t = .53, p = .60

Self-centered activities	64.66 (30.85) 0–100	69.01 (29.04) 0–100	**W = 32198.00**, **p = .035**	71.52 (27.76) 2–100	63.80 (31.29) 0–100	**t = 2.21**, **p = .29**

**SLEEP & SLEEPINESS**						

Sleepiness (ESS)	8.31 (4.01) 0–20	7.53 (4.38) 0–21	**W = 35712.50**, **p < .001**	6.94 (4.27) 0–19	8.18 (4.92) 0–22	**t = 2.90**, **p = .004**

Sleep quality PSQI-C1	1.15 (0.75) 0–3	1.37 (0.88) 0–3	**W = 7193.00**, **p < .001**	1.26 (0.90) 0–3	0.80 (0.94) 0–3	**W = 3554.00**, **p < .001**

Sleep latency PSQI-C2	1.09 (0.86) 0–3	1.29 (1.03) 0–3	**W = 7392.00**, **p < .001**	1.17 (0.91) 0–3	1.21 (0.89) 0–3	W = 2047.50, p = .71

Sleep duration PSQI-C3	0.38 (0.66) 0–3	0.34 (0.70) 0–3	W = 5114.00, p = .22	0.35 (0.66) 0–3	0.86 (0.85) 0–3	W = 606.00, p < .001

Sleep efficiency PSQI-C4	0.40 (0.74) 0–3	0.55 (0.85) 0–3	**W = 4588.50**, **p < .001**	0.53 (0.84) 0–3	0.70 (0.94)	W = 1138.50, p = .12

Sleep disturbance PSQI-C5	1.25 (0.50) 0–3	1.31 (0.55) 0–3	**W = 2520.50**, **p = .022**	1.32 (0.57) 0–3	2.18 (0.52) 0–3	**W = 409.50**, **p < .001**

Sleep pills PSQI-C6	0.51 (0.99) 0–3	0.54 (1.04) 0–3	W = 992.00, p = .34	0.47 (1.00) 0–3	0.53 (1.04) 0–3	W = 214.00, p = .51

Daytime dysfunction PSQI-C7	1.19 (0.95) 0–3	1.20 (0.99) 0–3	W = 10044.50, p = .27	1.16 (0.97) 0–3	1.03 (0.70) 0–3	W = 1419.00, p = .07

Naps	0.77 (1.31) 0–7.25	1.17 (1.81) 0–7.75	**W = 4932.50**, **p < .001**	1.11 (1.78) 0–7.25	0.61 (1.20) 0–6.50	**W = 1377.50**, **p = .011**


**Table 5:** Raw values and statistical outcomes of the paired sample t-tests/Wilcoxon tests (depending on the normality) for **retirees**.


	BEFORE LOCKDOWN (INITIAL SAMPLE, N = 124)	DURING LOCKDOWN (INITIAL SAMPLE, N = 124)	BEFORE VS DURING (INITIAL SAMPLE, N = 124)	DURING LOCKDOWN (FOLLOW-UP SAMPLE, N = 37)	1Y POST LOCKDOWN (FOLLOW-UP SAMPLE, N = 37)	DURING VS POST (FOLLOW-UP SAMPLE, N = 37)

	MEAN (SD) MIN-MAX	MEAN (SD) MIN-MAX		MEAN (SD) MIN-MAX	MEAN (SD) MIN-MAX	

**FATIGUE**						

Physical fatigue	8.70 (2.98) 4–18	9.41 (3.46) 4–17	**W = 1247.50**, **p = .003**	9.10 (3.23) 4–16	11.00 (3.45) 4–19	**t = –4.05**, **p < .001**

Mental Fatigue	8.61 (2.90) 4–15	9.15 (3.34) 4–19	**W = 1619.50**, **p = .041**	8.50 (3.20) 4–19	9.09 (3.00) 4–15	t = –1.415, p = .17

**ANXIETY STATE**						

General anxiety	–	11.44 (3.51) 6– 24	–	11.28 (3.35) 7–22	12.13 (26.56) 7–20	**W = 157.00**, **p = .027**

COVID-related anxiety	–	51.65 (29.76) 1–100	–	50.28 (26.36) 1–99	37.11 (29.00) 3–100	**t = 2.90**, **p = .006**

**LEISURE ACTIVITIES**						

Screen exposure, *min-24h*	128.08 (91.15) 0–420	173.19 (125.19) 0–660	**W = 57.00**, **p < .001**	186.49 (139.75) 5–660	144.98 (97.82) 3–378	t = 1.54, p = .13

Outdoor activities, *min-24h*	101.22 (82.66) 1–370	94.23 (94.50) 0–420	W = 2391, p = .17	93.11 (79.05) 10–360	69.58 (61.12) 0–300	t = 1.948, p = .059

Sports, *min-24h*	64.31 (63.74) 0–360	53.86 (64.42) 0–410	**W = 1859.50**, **p = .002**	45.87 (45.75) 0–150	42.03 (31.79) 0–120	t = 0.483, p = .63

**MENTAL LOAD**						

House duties	68.45 (27.26) 5–100	71.31 (27.85) 8–100	W = 2031.00, p = .21	72.25 (28.51) 8–100	73.16 (20.72) 4–100	t = –0.35, p = .73

Social interactions	79.60 (20.83) 20–100	71.96 (28.41) 1–100	**W = 3102.00**, **p < .001**	71.48 (27.84) 1–100	75.03 +–21.36 6–100	t = –0.77, p = .44

Self-centered activities	80.69 (19.78) 4–100	76.91 (23.58) 2–100	W = 3071.50, p = .90	76.68 (24.10) 2–100	77.92 +–22.09 21–100	t = –0.22, p = .82

**SLEEP & SLEEPINESS**						

Sleepiness (ESS)	7.29 (4.15) 0–20	6.75 (4.05) 0–20	**W = 1604.50**, **p = .008**	6.43 (3.62) 0–15	7.27 (4.23) 0–19	**t = –2.11**, **p = .041**

Sleep quality PSQI-C1	0.93 (0.63) 0–3	0.99 (0.70) 0–3	W = 112.50, p = .13	1.10 (0.71) 0–3	1.23 (0.66) 0–3	W = 48.00, p = .26

Sleep latency PSQI- C2	0.86 (0.74) 0–3	0.89 (0.81) 0–3	W = 132.00, p = .58	0.95 (0.78) 0–3	0.95 (0.78) 0–3	W = 45.50, p = 1.0

Sleep duration PSQI-C3	0.29 (0.64) 0–3	0.32 (0.69) 0–3	W = 32.50, p = .34	0.33 (0.66) 0–3	0.46 (0.68) 0–3	W = 10.00, p = .11

Sleep efficiency PSQI-C4	0.30 (0.58) 0–2	0.40 (0.70) 0–3	**W = 77.00**, **p = .042**	0.45 (0.71) 0–2	0.58 (0.68) 0–2	W = 48.00, p = .26

Sleep disturbance PSQI-C5	1.23 (0.48) 0–3	1.24 (0.50) 0–3	W = 72.00, p = .83	1.15 (0.48) 0–2	1.55 (0.60) 0–3	**W = 00.00** **p < .001**

Sleep pills PSQI-C6	0.58 (1.06) 0–3	0.61 (1.10) 0–3	W = 9.00, p = .18	0.70 (1.18) 0–3	0.83 (1.26) 0–3	W = 26.00 p = .56

Daytime dysfunction PSQI-C7	0.74 (0.61) 0–3	0.74 (0.65) 0–3	W = 297.00 p = 1.00	0.68 (0.70) 0–3	0.80 (0.46) 0–2	W = 28.00, p = .18

Naps	2.17 (2.62) 0–7.25	2.21 (2.66) 0–7.75	W = 189.00, p = .38	1.89 (2.54) 0–7.75	2.27 (2.38) 0–6.50	W = 173.50, p = .51


Fatigue. With the implementation of the lockdown, the subjective reports of physical and mental fatigues of both workers and retirees have both significantly increased.

Work- and leisure-related activities. The lockdown period was associated for workers with increased flexibility at work and less work activity per day, while the effort required by work did not change. For leisure activities, more time spent on screen and outdoor activities was observed for workers, while time allocated to sport did not change. Retirees reported more screen exposure time. However, the lockdown did not affect their time devoted to outdoor activities, although they significantly dedicated less time in doing sports.

Mental load. Wilcoxon’s tests revealed that the lockdown was associated in workers with a general increase in mental load for social interactions and work activities, while a decrease was observed in mental load related to house duties and self-centered activities. In retirees, an increase in mental load was observed only for social interactions.

Sleep and sleepiness. Workers’ level of sleepiness seemed to have significantly decreased with the lockdown, while the nap habits have increased. The lockdown has, however, induced a worst subjective quality of sleep (C1), a longer sleep latency (C2), a worst sleep efficiency (C4) as well as more sleep disturbances (C5) compared to the period before the mandatory containment. However, no effect was observed on sleep duration and use of sleep medicine. In contrast, retirees’ sleepiness has significantly decreased with lockdown while only their sleep efficiency (C4) has worsened. No significant change was observed concerning the retirees’ nap habits.

### Which daily life variables affect the evolution of fatigue levels during lockdown?

Linear regression models were performed to determine how change in fatigue level is explained by changes in variables that were found impacted by the COVID situation (see Table 6).

**Table 6:** Statistical outcome of the linear regression models seeking for associations between changes in physical and mental fatigue (dependent variables) and changes in demographics, anxiety state, work-related activities, leisure activities, mental load, sleep and sleepiness and their sub-scores **in (a) workers and (b) retirees** during lockdown.


VARIABLES	(a) WORKERS (N = 430)								(b) RETIREES (N = 130)							
	
	PHYSICAL FATIGUE				MENTAL FATIGUE				PHYSICAL FATIGUE				MENTAL FATIGUE			
			
	B (SE)	β	*t*	*p*	B (SE)	β	*t*	*p*	B (SE)	β	*t*	*p*	B (SE)	β	*t*	*p*

**DEMOGRAPHICS**																

Age	–0.04 (0.02)	–0.12	–2.43	**.02**	–0.02 (0.02)	–0.06	–1.10	.27	–0.02 (0.04)	–0.53	–0.55	.58	–0.02 (0.04)	–0.05	–0.53	.60

Gender (men)	0.70 (0.54)	0.06	1.30	.20	0.38 (0.57)	0.03	0.66	.51	0.23 (0.58)	0.04	0.40	.69	0.42 (0.52)	0.08	0.80	.43

Upper Secondary (vs Lower)	–6.03 (4.26)	–0.03	–1.42	.16	–1.40 (4.49)	–0.07	–0.31	.76	0.31 (1.79)	0.04	0.17	.86	0.95 (1.61)	0.15	0.59	.56

Bachelor (vs Lower Secondary)	–6.72 (4.20)	–0.69	–1.60	.11	–1.71 (4.42)	–0.17	–0.39	.70	0.57 (1.78)	0.10	0.32	.75	1.36 (1.60)	0.26	0.85	.40

Master (vs Lower Secondary)	–6.72 (4.19)	–0.74	–1.60	.11	–1.10 (4.42)	–0.12	–0.25	.80	1.07 (1.74)	0.18	0.62	.54	1.67 (1.56)	0.31	1.07	.29

**ANXIETY STATE**																

General anxiety	0.43 (0.05)	0.43	9.36	**<.001**	0.48 (0.05)	0.46	10.12	**<.001**	1.38 (0.48)	0.28	2.89	**.005**	1.16 (0.42)	0.27	2.77	**.01**

COVID-related anxiety	–0.02 (0.01)	–0.11	–2.42	**.02**	–0.02 (0.01)	–0.14	–3.02	**.003**	–0.0001 (0.01)	–0.001	–0.009	.99	0.01 (0.01)	0.11	1.16	.25

**WORK ACTIVITIES**																

Work from home (yes)	–0.15 (0.54)	–0.02	–0.28	.78	1.76 (0.57)	0.17	3.07	**.002**	–	–	–	–	–	–	–	–

Flexibility	0.01 (0.01)	0.12	2.38	**.02**	0.01 (0.01)	0.10	2.06	**.04**	–	–	–	–	–	–	–	–

Activity	0.001 (0.01)	0.01	0.14	.89	–0.01 (0.01)	–0.08	–1.47	.14	–	–	–	–	–	–	–	–

**LEISURE ACTIVITIES**																

Screen exposure	0.0001 (0.001)	0.004	–0.08	.94	0.002 (0.002)	0.06	1.14	.26	0.01 (0.004)	0.11	1.214	.23	0.001 (0.004)	0.03	0.31	.75

Outdoor activities	–0.01 (0.002)	–0.18	–3.54	**<.001**	–0.004 (0.002)	–0.09	–1.66	.098	–	–	–	–	–	–	–	–

Sports	–	–	–	–	–	–	–	–	–0.01 (0.004)	–0.29	–3.222	**.002**	–0.01 (0.004)	–0.13	–1.44	.15

**MENTAL LOAD**																

House duties	–0.01 (0.01)	–0.08	–1.60	.11	–0.01 (0.01)	–0.07	–1.45	.15	–	–	–	–	–	–	–	–

Social interactions	–0.03 (0.01)	–0.18	–3.78	**<.001**	–0.02 (0.01)	–0.16	–3.39	**.001**	–0.01 (0.01)	–0.05	–0.51	.61	–0.001 (0.01)	–0.006	–0.06	.95

Work	–0.02 (0.01)	–0.14	–3.22	**.001**	–0.03 (0.01)	–0.21	–4.85	**<.001**	–	–	–	–	–	–	–	–

Leisure	–0.03 (0.01)	–0.29	–5.68	**<.001**	–0.04 (0.01)	–0.32	–6.62	**<.001**	–	–	–	–	–	–	–	–

**SLEEP & SLEEPINESS**																

ESS	0.12 (0.06)	0.10	2.24	**.03**	0.17 (0.06)	0.14	3.05	**.002**	0.13 (0.13)	0.09	0.99	.33	0.16 (0.11)	0.13	1.43	.16

PSQI C1	1.54 (0.26)	0.34	5.86	**<.001**	1.44 (0.27)	0.30	5.28	**<.001**	–	–	–	–	–	–	–	–

PSQI C2	–0.05 (0.24)	–0.01	–0.19	.85	0.02 (0.25)	0.004	0.08	.94	–	–	–	–	–	–	–	–

PSQI C4	0.64 (0.23)	0.13	2.74	**.01**	0.62 (0.24)	0.12	2.57	**.01**	–0.35 (0.49)	–0.06	–0.71	.48	–0.16 (0.45)	–0.03	–0.37	.71

PSQI C5	–0.11 (0.39)	–0.01	–0.29	.78	0.64 (0.40)	0.08	1.59	.11	–	–	–	–	–	–	–	–

Naps	–0.01 (0.11)	–0.004	–0.09	.93	0.02 (0.11)	0.01	0.21	.83	–	–	–	–	–	–	–	–


*Note*. **B**: unstandardized coefficient estimates. **SE**: Standard errors. β: standardized coefficient estimates. ***t***: *t*-value for testing the null hypothesis. ***p***: *p*-value.

Demographics. Among workers, we observed a significant negative association between age and changes in physical fatigue, showing that younger adults were more physically fatigued. No other association between demographic components (gender and educational level) and fatigue have been observed for workers nor retirees.

Anxiety state. Workers’ and retirees’ general anxiety state during lockdown was positively associated with both changes in mental and physical fatigue (with higher anxiety associated with a higher increase of fatigue from before to during lockdown). For workers, changes in COVID-related anxiety were negatively associated with the changes in these two types of fatigue (with people being more afraid of the virus being less fatigued).

Work- and leisure-related activities. In workers, linear regression analyses highlighted a positive association between changes in both types of fatigue and flexibility at work (more flexibility at work is associated with more fatigue). Working from home was also positively associated with significant increases in mental fatigue. Concerning leisure-related activities, changes in outdoor activities were negatively associated with physical fatigue in the workers cohort (more outdoors activities linked to less physical fatigue). For retirees, linear regression analyses showed that changes in time dedicated to sports were negatively associated with changes in physical fatigue, meaning that reducing sport activity is associated with more physical fatigue. No other significant associations were found between the daily activities’ sub-components and fatigue.

Mental load. In the working population, linear regression analyses showed that the increase in mental load needed in social interactions, work and leisure activities was associated with an increase of both mental and physical fatigue. These results indicate that the higher the mental load becomes by comparison to the pre-lockdown situation, the higher is the increase in feeling of fatigue during the lockdown. Conversely, no significant associations were found between fatigue and the mental load’s components in the retired cohort.

Sleep and sleepiness. Changes in sleepiness, subjective quality of sleep (C1) and sleep efficiency (C4) were revealed to be the components negatively associated with mental and physical fatigue in workers. More precisely, less fatigue was associated with a better sleep and less sleepiness. Considering the retired cohort, no significant associations have been found between fatigue and sleep components.

### Data set 2: Score comparison: during the lockdown and one-year later

Data set 2 explored the difference in daily habits and psychological aspects and their impact on both mental and physical fatigue of workers and retirees during the COVID-19 lockdown compared to one-year post-lockdown, which covers the period from March 2021 to April 2021. Raw data and results of statistical tests are presented in Tables 4 and 5. To ensure that the subsample that completed the survey at one-year follow-up did not differ from the whole sample at baseline, demographic variables and scores reported before and during lockdown in 2020 were compared between the two groups. We only observed a lower anxiety level in 2020 in the subsample that completed the survey one year later (mean = 12.11, SD = 3.80) compared to the whole baseline sample (mean = 13.07, SD = 4.23) (*p* = .02) (see Appendix F for presentation of all comparisons).

Fatigue. Physical fatigue has significantly increased for workers and retirees from during the lockdown restrictions of March-May 2020 to one year after that period, which was not subject to lockdown anymore. No effect was found for mental fatigue for either of our two populations.

Anxiety state. The level of general anxiety of the participants was compared at the two time points. An increase in general anxiety level was observed while the COVID-related anxiety decreased one-year post-lockdown in both workers and retirees.

Work- and leisure-related activities. One-year post-lockdown, workers have significantly less teleworked, had less work flexibility and were busier during a workday. With regard to leisure activities, workers have spent less time in outdoor activities after lockdown compared to during the lockdown, with no change for time dedicated to screen exposure and sports. On the other hand, retirees did not change the time dedicated to any of their daily activities.

Effect of mental load. Mental load of workers required for house duties and self-centered activities has increased with the end of the lockdown. For retirees, no difference was found between the two time periods.

Sleep and sleepiness. Compared to during the lockdown period, workers’ sleepiness, sleep duration (C3), and sleep disturbances (C5) have significantly increased, while their nap score has decreased. Also, workers reported a better subjective quality of sleep (C1) than during lockdown. Retirees only showed increased sleepiness and sleep disturbances.

### Which daily life variables affect the increased fatigue level after lockdown?

Linear regression models were performed to determine how change in fatigue levels is explained by changes in variables that were found impacted by the March 2020 situation one year after lockdown (see Table 7). The analyses were also performed on the delta score for mental fatigue, as we were interested to determine if the same variables affect mental fatigue after the lockdown.

**Table 7:** Explanatory statistical outcome of the linear regression models seeking for associations between changes in physical and mental fatigue (dependent variables) and changes in demographics, anxiety state, work-related activities, leisure activities, mental load, sleep and sleepiness and their sub-scores in (a) workers and (b) retirees one-year post-lockdown.


VARIABLES	(a) WORKERS (N = 133)								(b) RETIREES (N = 40)							
	
	PHYSICAL FATIGUE				MENTAL FATIGUE				PHYSICAL FATIGUE				MENTAL FATIGUE			
			
B (SE)	β	*t*	*p*	B (SE)	β	*t*	*p*	B (SE)	β	*t*	*p*	B (SE)	β	*t*	*p*

**DEMOGRAPHICS**																

Age	–0.001 (0.03)	–0.004	–0.05	.96	0.02 (0.04)	0.06	0.66	.51	–0.07 (0.10)	–0.14	–0.76	.45	0.07 (0.08)	0.15	0.89	.38

Gender (men)	–1.12 (0.80)	–0.13	–1.41	.16	0.17 (0.92)	0.02	0.18	.86	0.91 (1.04)	0.15	0.88	.39	1.45 (0.86)	0.27	1.69	.10

Upper Secondary (vs Lower)	–	–	–	–	–	–	–	–	0.01 (3.57)	0.001	0.002	1.00	–0.70 (2.94)	–0.09	–0.24	.82

Bachelor (vs Lower Secondary)	1.24 (1.63)	0.15	0.76	.45	–0.79 (1.90)	–0.08	–.41	.68	–0.50 (3.34)	–0.08	–0.15	.88	–0.49 (2.75)	–0.09	–0.18	.86

Master (vs Lower Secondary)	1.50 (1.58)	0.19	1.58	.34	–1.30 (1.82)	–0.14	–0.71	.48	–1.60 (3.47)	–0.26	–0.46	.65	–2.71 (2.86)	–0.49	–0.95	.35

**ANXIETY STATE**																

General anxiety	0.29 (0.08)	0.33	3.79	**<.001**	0.35 (0.09)	0.34	3.98	**<.001**	0.05 (0.18)	0.05	0.30	.77	0.19 (0.16)	0.20	1.18	.25

COVID-related anxiety	–0.01 (0.01)	–0.13	–1.48	.14	–0.02 (0.01)	–0.16	–1.81	.07	–0.01 (0.02)	–0.11	–0.64	.53	–0.001	–0.01	–0.07	.94

**WORK ACTIVITIES**																

Work from home (yes)	0.67 (0.74)	0.09	0.91	.37	–0.93 (0.84)	–0.11	–1.11	.27	–	–	–	–	–	–	–	–

Flexibility	–0.01 (0.01)	–0.06	–0.64	.53	.02 (0.01)	0.15	1.62	.11	–	–	–	–	–	–	–	–

Activity	–0.02 (0.01)	–0.19	–2.00	**.05**	–0.03 (0.01)	–0.22	–2.40	**.02**	–	–	–	–	–	–	–	–

**LEISURE ACTIVITIES**																

Outdoor activities	–0.001 (0.00)	–0.13	–1.46	.15	–0.001 (0.00)	–0.03	–0.36	.72	–	–	–	–	–	–	–	–

MENTAL LOAD																

House duties	–0.01 (0.01)	–0.09	–0.92	.36	–0.006 (0.01)	–0.06	–0.60	.55	–	–	–	–	–	–	–	–

Leisure	0.01 (0.01)	0.10	1.01	.31	–0.002 (0.01)	–0.02	–0.19	.85	–	–	–	–	–	–	–	–

**SLEEP & SLEEPINESS**																

ESS	–0.01 (0.07)	–0.01	–0.12	.91	–0.07 (0.08)	–0.08	–0.87	.39	0.18 (0.19)	0.16	0.97	.34	0.08 (0.17)	0.07	0.44	.66

PSQI C1	0.16 (0.25)	0.06	0.64	.53	0.53 (0.29)	0.17	1.84	.07	–	–	–	–	–	–	–	–

PSQI C3	–0.02 (0.33)	–0.006	–0.06	.95	–0.05 (0.37)	–0.01	–0.14	.89	–	–	–	–	–	–	–	–

PSQI C5	0.57 (0.53)	0.10	1.09	.28	0.98 (0.60)	0.15	1.63	.11	0.93 (0.96)	0.16	.97	.34	0.65 (0.87)	0.12	0.75	.46

Naps	0.22 (0.17)	0.13	1.32	.19	–0.05 (0.19)	–0.02	–0.25	.80	–	–	–	–	–	–	–	–


*Note*. **B**: unstandardized coefficient estimates. **SE**: Standard errors. β: standardized coefficient estimates. ***t***: *t*-value for testing the null hypothesis. ***p***: *p*-value.

**Demographics**. In both workers and retirees, the linear regression model analysis revealed no association between demographic characteristics (age, gender, educational level) and the evolution of fatigue.

**Anxiety state**. The increase of general anxiety state of workers 1-year post lockdown was positively associated with changes in both physical and mental fatigue. Conversely, no association was found for retired people between anxiety state variables and fatigue.

**Work- and leisure-related activities**. In workers, our linear regression models revealed that changes in the degree of occupation at work, – in other words, how busy a worker becomes in 2021 compared with 2020 -, were negatively associated with both types of fatigue (meaning that higher degree of occupation is linked to lower levels of mental and physical fatigue from lockdown to 1-year later). No association between workers’ outdoor activities time and fatigue was highlighted during the regression model analysis.

**Mental load**. No significant association between changes in mental load variables between 2020 and 2021 and changes in fatigue level was found.

**Sleep and sleepiness**. Linear regression analyses demonstrated no association between any sleep characteristics and fatigue changes between and after lockdown, nor in workers neither in retirees.

## Discussion

This study investigated how both physical and mental fatigue have evolved with the implementation of the COVID-19 lockdown in Belgium as well as how their evolution is associated and modulated with several spheres of daily life and psychological factors namely: demographics, anxiety state, leisure-related activities, sleep and sleepiness, investment at work, and mental load.

### Are physical and mental fatigue different entities?

As expected, and in line with previous studies (Torrente et al., 2022), we have demonstrated that the March 2020 lockdown context was characterized by increased feelings of physical and mental fatigue among both workers and retirees. Moreover, specificities in the variables associated with each kind of fatigue were highlighted, consistent with the idea that physical and mental fatigue are based on two distinct constructs. The literature is currently debating the degree of overlap between physical and mental fatigue, and how they affect activities depending on their type (Boolani & Manierre, 2019; Cella & Chalder, 2010; De Raaf et al., 2013). Indeed, it is hypothesized that mental fatigue will exert a greater influence on activities linked to cognitive performance, while physical fatigue will primarily impact activities associated with physical performance (Barker Steege & Nussbaum, 2013). The idea that each type of fatigue is preferentially associated with specific activities over others is challenged as mental fatigue may also have adverse effects on physical performance (Kuppuswamy, 2017; Martin et al., 2018; McMorris, 2020; McMorris et al., 2018; Mehta & Parasuraman, 2014; Van Cutsem et al., 2017). Here, we have a natural setting to investigate whether changes in life habits and other variables due to the lockdown have affected levels of physical and mental fatigue differently. Based on data set 1 (workers), we can observe that both types of fatigue frequently, but not always, associate with the same activities. Exceptions remain regarding age and outdoor activities which are specific to physical fatigue, and regarding telework context specific to mental fatigue. Our results provide additional support to the claim that fatigue should be considered as a multidimensional concept, although unique characteristics of types of fatigue can be observed.

### Both types of fatigue are linked to anxiety state

Consistent with previous research (Abdul Rashid et al., 2023; Glowacz & Schmits, 2020; Morin et al., 2022; Ramiz et al., 2021; Torrente et al., 2022), the extraordinary situation provoked higher anxiety state among the population, which was related to higher physical and mental fatigue. Beyond any period of health crisis, links between anxiety state and fatigue have been extensively studied, revealing a comorbid relationship between anxiety and fatigue, suggesting overlapping between the two entities, and reinforcing the need to address anxiety and fatigue simultaneously (Lamers et al., 2013; Williams et al., 2021). Furthermore, the COVID-19 Mental Disorders Collaborators (2021) observed a 25% increase of anxiety disorders during the COVID-19 period (Santomauro et al., 2021), while anxiety has been revealed to be the most impacted aspect of mental health due to the pandemic (Hampshire et al., 2021). Interestingly, and in line with a previous study (Pieh et al., 2021), we have found that the anxiety level of our population persisted and even kept increasing one-year after the lifting of lockdown measures. However, the association we observed between fatigue and anxiety level in workers and retirees remains significant one year post lockdown in workers only. Our data does not allow a clear interpretation of the divergence between the two groups. However, we may suggest that employment vs retirement influences the association between fatigue and anxiety in our sample (see below for a discussion about fatigue and aging).

### Influence of life characteristics on fatigue across the pandemic context

Other life characteristics related to fatigue such as mental load, work and sleep appear to fluctuate across time in response to the progression of the pandemic.

First, higher mental load in social interactions, work and leisure activities was revealed to be associated with higher mental and physical fatigue. Even though the direction of this relation cannot be revealed in our analysis, it is well established that higher mental load leads to greater fatigue state. For instance, engaging in cognitively demanding tasks eventually leads to the development of fatigue (Borragán et al., 2017). Only house duties were found not to be associated with fatigue. This can be explained by greater routine nature of this activity compared to others, requiring less planification during its realization.

As mental load is known to influence fatigue levels, disturbed sleep is another significant predictor of fatigue (Åkerstedt et al., 2004). Specifically, we found that the lockdown onset considerably affected the sleep patterns of the working population. Participants reported worsened sleep quality, longer time to fall asleep, decreased sleep efficiency, more sleep disturbances, and increased scores of napping. Previous studies (Beck et al., 2021; Cellini et al., 2021; Morin et al., 2022) have also reported similar findings of increased sleep disturbances and poorer sleep quality during lockdown compared to the pre-pandemic period. Furthermore, we observed that subjective measures of sleep (sleep quality and efficiency) were associated with the evolution of fatigue in the worker cohort during the restrictions. Even though no associations with fatigue persisted, sleep disturbance, in addition to anxiety, kept being worsened even one-year after the lockdown onset for workers and retirees.

As previously mentioned, sleep and work are key factors in the development of fatigue for workers (Åkerstedt et al., 2004). During the pandemic context, social gatherings and activities were not allowed, and workers thus needed to quickly adapt. Consequently, new tools emerged including online meetings and workers rapidly adopted new habits regarding their professional life such as working from home or making flexible working hours. This could in turn contribute to the development of fatigue (Lestari & Fayasari, 2022; Oducado et al., 2022). Here, approximately 73% of our worker cohort teleworked during the lockdown. Our participants reported a decrease of job workload and greater flexibility in occupational duties. These results are in line with changes in workstyles mentioned in the literature (Anderson & Kelliher, 2020; H. Chung, 2022; Spurk & Straub, 2020). Here, these changes, as well as work-related mental load, were associated with a higher mental fatigue state, highlighting the adverse consequences of mandatory restrictions on workers. In our follow-up study, we observed a decrease in the frequency of teleworking and the level of flexibility experienced by individuals, alongside an increase in reported daily work volume. Interestingly, we found that employees who had higher workload experienced lower levels of physical and mental fatigue one year after the initial lockdown.

As a whole, these results suggest a complex interplay between work-related variables associated with a fatigue state. Particularly, maintaining a sense of productivity and commitment at work during the pandemic may contribute to enhanced well-being and serve as a strategy to alleviate pandemic fatigue through motivational factors. Moreover, the observation of fewer associations between fatigue and work characteristics at the one-year follow-up suggests that employees may have gradually adapted to their new work situation. This highlights the potential of returning to pre-pandemic working habits to reduce previously generated fatigue. Potential risk factors inducing exacerbated feeling of mental fatigue may be difficulties in separating work and personal life as induced by teleworking (Borowiec & Drygas, 2022; Fan & Smith, 2018) and a reduced cognitive functioning provoked by the lockdown-related changes in the work condition (Fiorenzato et al., 2021). It was previously shown that such situation could directly lead to a burnout syndrome if no considering measures are taken (Demerouti et al., 2002; Ekstedt et al., 2006). Identifying risk factors specific to lockdown and pandemic situation may be useful to implement prevention strategies.

### Retirement-related effects of fatigue

In the case of retirees, unlike workers, restrictions did not appear to be associated with changes in sleep metrics. In a more general way, the daily activities of retirees were less impacted by the pandemic. Even though no statistical comparison between workers and retirees can be made regarding the impact of lockdown, it seems that workers may be more vulnerable to the development of fatigue, as more aspects of their lives were linked to fatigue evolution. The absence of significant correlations between sleep habits, daily activities, and fatigue evolution among retirees at one year post lockdown suggests that fatigue may have arisen primarily from the changes in daily life resulting from the lockdown. This observation may be attributed to the retiree’s lack of active work engagement. Of note, retirees were older than workers, but the data do not allow to separate the effects due to work versus retirement and the effects of age.

### Fatigue emerged from the pandemic situation rather than the lockdown

Pandemic context can be seen as a “natural laboratory” that allow us to observe spontaneously the effect of habit changes and stress on fatigue. With this study, we have demonstrated that the stay-at-home confinement due to COVID-19 pandemic has induced changes in everyday life in both workers and retirees. Furthermore, it seems that these changes mostly compromised the level of fatigue, anxiety state, and sleep habits of the population. Our one-year follow-up study highlighted that these changes were not restricted to the lockdown period *per se*. Consequently, the changes observed here may also originate from the health situation or post-lockdown lifestyle adjustments. Living through a pandemic seems to trigger a series of upheavals in the population resulting in increased mental and physical fatigue, qualified by some authors by “lockdown fatigue” (Labrague & Ballad, 2021). Our study suggests that this *lockdown fatigue* emerges due to disruptions in daily routines (including work habits) following social isolation. However, the increase of fatigue levels measured during the lockdown did not resume its baseline one year after the lockdown ended. On the contrary, physical fatigue continued to increase significantly compared to the pre-lockdown period (< March 2020). Talking about *lockdown fatigue* is therefore delicate since fatigue would emerge from the general health situation rather than from the implementation of the lockdown. Our findings are consistent with the ones found by Moradian et al. (2021) which highlighted the prolonged negative impact of restrictions on the German population, and in which fatigue is interpreted as “pandemic fatigue”. In conclusion, fatigue persisted after lockdown (and even increased for physical fatigue). Interestingly, the anxiety level (excluding COVID-related anxiety) has exhibited identical upward trend within both groups. Additionally, only a minimal number of associations with daily activities, sleep, and mental load remained post-lockdown. Consequently, it prompts the question: Does fatigue originate from the same underlying cause during both the lockdown period and one year later? During the lockdown, fatigue could potentially be linked to changes in lifestyle habits and mental load. However, one year later, it appears to be primarily driven by the anxiety state, given that the association with mental load has diminished. Unfortunately, direct assessment of this relationship cannot be assessed within the scope of this study.

### Limitations and perspectives

Our study has several limitations that should be acknowledged and considered for future research. Firstly, the use of online questionnaires introduces self-report bias, particularly recall bias, as participants were required to recall their pre-pandemic situation while already enrolled in a lockdown context. Secondly, we were unable to directly assess the relationship between the lifestyle variables within the scope of this study, due to lack of statistical power. However, our results seem to show that fatigue emerges from disruptions in lifestyle habits.

Third, the disparity in sample size between the two age groups prevents comparison between them. Another limitation pertains to the disproportionate representation of females compared to males within the workers group. Despite efforts to recruit a diverse participant cohort, inherent biases in research participation resulted in a higher engagement of females, leading to a skewed gender distribution (Dickinson et al., 2012; Mohajer & Jan, 2019). Expanding research on the differential impact of lifestyle changes and their relationship with fatigue in males and females would significantly contribute to a more comprehensive understanding of these phenomena. Indeed, some studies (Avlund, 2010; Butt et al., 2010) have found that women tend to experience more fatigue than men, while others (Watt, 2000; Wylie et al., 2022) did not showed any gender-difference (for specific discussion of gender-related fatigue in the context of COVID-19, see Rudroff et al., 2020, 2022). Fourth, we experienced a significant attrition of participants during the follow-up phase. Therefore, our explanatory follow-up analysis should be interpreted carefully.

Moreover, an unexpected observation was noted regarding lower anxiety levels in the subsample that completed the survey one year later compared to the entire baseline sample. This observation could potentially affect our results slightly, and while intriguing, we lack a definitive explanation for this discrepancy in anxiety levels. Addressing this discrepancy and understanding the potential reasons behind it requires further exploration. Yet, it is crucial to acknowledge that this variation in anxiety levels could also stem from random chance or variability inherent within the sample, likely indicative of sampling bias. Finally, our survey does not include reports of COVID-19 unrelated events between 2020–2021 that may affect fatigue, anxiety levels, and lifestyle (such as wedding, birth of a child, death of a relative, major occupational changes…). However, such events should randomly intervene between participants and consequently not significantly affect the results.

Finally, we stated in the method section that the study was not pre-registered. We acknowledge this as a major limitation that complicates assessing reporting bias that may be associated with the study protocol or data analysis.

Addressing the gaps identified in our study, future research could significantly benefit from incorporating objective measures of fatigue to better understand its relationships with daily life characteristics and well-being. The administration of cognitive tests as objective measures of fatigue could offer valuable insights into fatigability dynamics (Hassan et al., 2023). Moreover, recent studies have showcased promising means in assessing fatigue using physiological measures as pupillometry (Gergelyfi et al., 2015; Guillemin et al., 2022). This non-invasive technique holds advantage as it captures a natural phenomenon (the pupillary response), which has been demonstrated to be modulated by fatigue (Bafna & Hansen, 2021; Morad et al., 2000).

## Conclusion

In conclusion, our study conducted during the COVID-19 pandemic demonstrated that the stay-at-home confinement and habit changes brought significant alterations in the lives of both workers and retirees. These changes notably impacted fatigue levels, anxiety state, and sleep habits. Importantly, fatigue levels did not return to baseline even one year after the lockdown ended. Although no causality can be established in our study, and that a bidirectional relationship cannot be excluded for the previously attempted explanations, our study has served to highlight the importance of preventing increased fatigue in the population in a context of pandemic, and aimed to encourage guidelines that can be put in place to best manage the inherent negative effects of fatigue. However, the observations made in this exploratory study may provide a foundational basis for future studies with specific hypotheses regarding the associations between the variables under investigation.

## Data Accessibility Statement

Data and analysis code are openly available at the project’s Open Science Framework page (https://osf.io/4ef2s/?view_only=1ecf3adac21944e68612a0b07dbe363b). Earlier version of this work was presented at the 2022 Annual Meeting of the Belgian Association for Psychological Sciences (BAPS), Leuven, Belgium.

## Additional File

The additional file for this article can be found as follows:

10.5334/pb.1259.s1 Supplementary Material. Appendix A–Appendix F.
