# Supplementary material for: The Influence of Changes in Daily Life Habits and Well-Being on Fatigue Level During COVID-19 Pandemic
Source: Psychol Belg. 2024 Jul 18;64(1):85–107. doi: 10.5334/pb.1259 (PMC11259104; doi:10.5334/pb.1259)
Supplement: Supplementary Material. — Appendix A–Appendix F. [file pb-64-1-1259-s1.pdf]

Appendix A

Items examples

ANXIETY STATE

- *General anxiety:* I feel upset

- *COVID-related anxiety:* In the current context, how do you rate your level of fear?

WORK-RELATED ACTIVITIES

- *Job effort:* How do you rate the level of effort required for you to perform your work currently?

- *Work schedule flexibility:* What is the degree of flexibility of your work schedule currently?

- *How professionally busy at work:* To what extent are you professionally occupied currently?

LEISURE-RELATED ACTIVITIES

- *Sports:* How long do you engage in physical activity? (minutes/day)

- *Outdoor activities:* How much time do you spend outdoors? (minutes/day)

- *Use of screen devices:* How much time do you spend in front of screens (for leisure and work)? (minutes/day)

MENTAL LOAD

In the past few days, I have felt mentally available to:

- *House duties*: Perform household tasks

- *Social interaction:* Have a constructive interaction with a loved one

- *Work:* Focus on my work

- *Self-centered leisure activities:* Engage in activities for myself, dedicate time to my hobbies (reading a book, watching a movie, sports, video games, etc.)

SLEEP QUALITY AND SLEEPINESS

- *PSQI:* How many hours of actual sleep do you get each night?

- *ESS:* In the past week, what was the likelihood of you dozing off (gradually falling asleep, half-asleep) sitting quietly after a meal without alcohol

- *Nap:* Do you take naps? How often do you take naps? How long are your naps on average?

FATIGUE

- *MFI physical:* Physically, I feel in excellent shape

- *MFI mental:* It took a lot of effort for me to concentrate

Appendix B

Nap score

A nap score was calculated based on the quantity of naps per week (ranging from 1 “less than once a week” to 7 “more than once a day”) and their duration (ranging from 0 “less than 10 minutes” to 1 “more than 2 hours”). Total nap score is the sum of the scores obtained for quantity and duration, ranging from 1 to 8.

Table B1

Detailed nap score

**Quantity of naps per week**	**Duration of naps per week**
Less than once a week: score of 1	< 10 min: score of 0
1-2 times a week: score of 2	10-30min: score of 0.25
3-4 times a week: score of 3	30-60min: score of 0.5
4-5 times a week: score of 4	1-2 hours: score of 0.75
5-6 times a week: score of 5	> 2h: score of 1
Once a day: score of 6	
More than once a day: score of 7	

Appendix C

Detailed results on the evolution of fatigue across the three time-points

We used mixed effect model analyses to examine the evolution of mental and physical fatigue in our two groups: workers and retirees. Tukey post-hoc tests with adjusted p-values were employed when significant effects (*p* <.05) were identified.

Regarding physical fatigue, we observed a significant group effect (F(1,693.8) = 8.93, *p* =.003) and a significant session effect (F(2,794.4) = 31.43, *p* < .001), but no significant interaction effect (F(2,794.4) = 0.74, *p* =.48). Specifically, retirees reported less physical fatigue than workers (t(693.8) = -2.99, *p* < .003). Moreover, physical fatigue was higher post-lockdown compared to pre-lockdown (t(854.8) = 7.63, *p* < .001) and during lockdown (t(854.8) = 4.72, *p* < .001), and higher during the lockdown compared to pre-lockdown (t(726.9) = 4.50, *p* < .001).

For mental fatigue, our analyses yielded significant effects for group (F(1,692.1) = 18.71, p < .001), session (F(2,793.9) = 21.82, p < .001) and the interaction between group and session (F(1,793.9) = 6.81, p = .001). Retirees reported less mental fatigue than workers (t(692.1) = -4.33, *p* < .001). Moreover, fatigue increased post-lockdown compared to pre-lockdown (t(858.5) = 3.80, *p* < .001), and during the lockdown compared to pre-lockdown (t(722.2) = 6.34, *p* < .001). Finally, post-hoc tests on the interaction effect showed that workers experienced more mental fatigue during the lockdown compared to pre-lockdown (t(722.2) = 10.60, *p* < .001), and were less fatigued before lockdown compared to post-lockdown (t(862.3) = -4.99, *p* < .001). There was no difference across sessions in the group of retirees.

Table C1

Raw values of the statistical outcome of the mixed effect model analyses

	**Workers (N = 133)**		**Retirees (N = 40)**	
	**Physical**	**Mental**	**Physical**	**Mental**

	**Mean (SD)**<br>**Min-Max**	**Mean (SD)**<br>**Min-Max**	**Mean (SD)**<br>**Min-Max**	**Mean (SD)**<br>**Min-Max**
**Before**	9.84 (3.68)<br>4 -20	8.95 (3.14)<br>4 -19	8.47 (2.51)<br>4 - 14	7.92 (2.63)<br>4 - 15
**During**	10.02 (3.53)<br>4 - 20	10.82 (3.98)<br>4 - 20	9.10 (3.23)<br>4 - 16	8.50 (3.20)<br>4 - 19
**Post**	11.66 (4.10)<br>4 - 20	10.58 (4.11)<br>4 - 20	11.00 (3.45)<br>4 - 19	9.09 (3.00)<br>4 - 15

Appendix D

Model summaries of the linear regressions

Table D1

Model summaries of the linear regression models seeking for associations between changes in physical and mental fatigue (dependent variables) and changes in demographics, anxiety state, work-related activities, leisure activities, mental load, sleep and sleepiness and their sub-scores ***in (a) workers and (b) retirees*** *during lockdown*

	(a) Workers						(b) Retirees					
	Physical Fatigue			Mental Fatigue			Physical Fatigue			Mental Fatigue		
**Variables**	Adjusted R^2	*F* (d)	*p*	Adjusted R^2	*F* (d)	*p*	Adjusted R^2	*F* (d)	*p*	Adjusted R^2	*F* (d)	*p*
**DEMOGRAPHICS**	0.011	1.92 (5,416)	.09	-0.003	1.92 (5,416)	.59	-0.026	0.384 (5,115)	.86	-0.018	0.582	.713
**ANXIETY STATE**	0.167	43.86 (2,427)	**<.001**	0.190	51.16 (2,427)	**<.001**	0.065	5.178 (2,118)	**.007**	0.096	7.389 (2,118)	**<.001**
**WORK ACTIVITIES**	0.007	1.976 (3,397)	.117	0.024	4.266 (3,397)	**.006**	-	-	-	-	-	-
**LEISURE ACTIVITIES**	0.028	7.118 (2,419)	**<.001**	0.009	2.938 (2,419)	.054	0.086	6.527 (2,115)	**.002**	0.003	1.156 (2,115)	.32
**MENTAL LOAD**	0.245	35.174 (4,418)	**<.001**	0.292	44.546 (4,418)	**<.001**	-0.006	0.263 (1,120)	.61	-0.008	0.004 (1,120)	.95
**SLEEP & SLEEPINESS**	0.160	14.64 (6,423)	**<.001**	0.179	16.558 (6,423)	**<.001**	-0.004	0.738 (2,121)	.48	0.001	1.091 (2,121)	.34

Note. Model summary of the H_1. **Adjusted R^2**: proportion of variance in the dependent variable that is explained by the independent variables. ***F***: F-value. **d**: numerator and denominator degrees of freedom of F change. ***p***: *p*-value.

Table D2

Model summaries of the linear regression models seeking for associations between changes in physical and mental fatigue (dependent variables) and changes in demographics, anxiety state, work-related activities, leisure activities, mental load, sleep and sleepiness and their sub-scores ***in (a) workers and (b) retirees*** *one-year post-lockdown*

	**(a) Workers**						**(b) Retirees**					
	**Physical Fatigue**			**Mental Fatigue**			**Physical Fatigue**			**Mental Fatigue**		
**Variables**	Adjusted R^2	*F* (d)	*p*	Adjusted R^2	*F* (d)	*p*	Adjusted R^2	*F* (d)	*p*	Adjusted R^2	*F* (d)	*p*
**DEMOGRAPHICS**	-0.005	0.830 (4,123)	.51	-0.020	0.391 (4,123)	.82	-0.040	0.699 (5,34)	.63	0.114	2.007 (5,34)	.10
**ANXIETY STATE**	0.093	7.489 (2,125)	**<.001**	0.106	8.533 (2,125)	**<.001**	-0.043	0.238 (2.35)	.79	-0.017	0.695 (2,35)	.51
**WORK ACTIVITIES**	0.028	2.160 (3,119)	.10	0.045	2.909 (3,119)	**.04**	-	-	-	-	-	-
**LEISURE ACTIVITIES**	0.009	2.124 (1,125)	.15	-0.007	0.132 (1,125)	.72	-	-	-	-	-	-
**MENTAL LOAD**	-0.005	0.70 (2,125)	.50	-0.012	0.268 (2,125)	.77	-	-	-	-	-	-
**SLEEP & SLEEPINESS**	-0.011	0.719 (5,123)	.61	0.030	1.783 (5,123)	.12	0.001	1.017 (2,37)	.37	-0.031	0.407 (2,37)	.67

Note. Model summary of the H_1. **Adjusted R^2**: proportion of variance in the dependent variable that is explained by the independent variables. ***F***: F-value. **d**: numerator and denominator degrees of freedom of F change. ***p***: *p*-value.

Appendix E

Data Transparency

The data reported in this manuscript have been previously published to some extent and were collected as part of a larger data collection. The findings from the data set 1 collection, which were acquired during the lockdown in 2020, have been reported in separate manuscripts: MS1 (published in Cellini et al., 2021), which focuses on sleep data (N = 650), MS2 (published in Folville et al. (2023), which focuses on autobiographical memory data (N = 225), and MS3 (current), which focuses on fatigue and lifestyle variables. The table below provides an overview of the inclusion of each data variable from data set 1 in each study, as well as the current status of each study. The variables acquired for data set 2, which was collected one year after the lockdown, have only been published in the present manuscript (MS3). These variables are identical to those acquired for data set 1.

Table E1

Overview of Data Variables in Studies

Variables in the Complete Data Set 1	MS1 (published)	MS 2 (published)	MS 3 (current)
Demographics			
Age		X	X
Gender		X	X
Educational level			X
Working status	X		X
Psycho-affective variables			
Mood	X	X	
Stress	X	X	
STAI		X	X

EMMBEP		X	
OQ45		X	
Memory variables			
Clarity		X	
Richness of visual, sensory details		X	
Precision of location, time		X	
People		X	
Objects		X	
Emotions/thoughts		X	
Reliving		X	
Emotional intensity		X	
Emotion valence		X	
Experiential diversity			
Valorizing activities		X	
Satisfactory activities		X	
Diversity of activities		X	
Novelty of activities		X	
Days look alike		X	
Work-related variables			
Effort			X
Flexibility			X
Activity			
Employment status	X		
Working from home	X		X
Sleep and sleepiness variables			
PSQI total score	X	X	X
PSQI sub-components			X

Bedtime	X		
Risetime	X		
Hours spent in bed	X		
Sleep duration	X		
Sleep onset latency	X		
Sleep midpoint	X		
Use of medication for sleeping	X		
Naps			X
ESS			X
Dream valence		X	
Activities-related variables			
Screen time		X	X
Quantity of social interactions		X	
Outdoor activities time			X
Sports time			X
Fatigue			
Mental fatigue			X
Physical fatigue			X
Mental load			
House duties			X
Social interactions			X
At work			X
Self-centered activities			X
COVID-19 questions			
COVID-19 news		X	
COVID-19 chats		X	
Previous infection	X		

Fear of infection	X	X	X

References

MS 1 : Cellini, N., Conte, F., De Rosa, O., Giganti, F., Malloggi, S., Reyt, M., Guillemin, C., Schmidt, C., Muto, V., & Ficca, G. (2021). Changes in sleep timing and subjective sleep quality during the COVID-19 lockdown in Italy and Belgium : Age, gender and working status as modulating factors. *Sleep Medicine*, *77*, 112-119. https://doi.org/10.1016/j.sleep.2020.11.027

MS 2: Folville, A., Willems, S., Cheriet, N., Geurten, M., Guillemin, C., Muto, V., Requier, F., Reyt, M., Collette, F., Schmidt, C., & Bastin, C. (2023). Well-being during COVID-19-related first lockdown : Relationship with autobiographical memory and experiential diversity. *Applied Cognitive Psychology*, *37*(5), 1059-1070. https://doi.org/10.1002/acp.4104

Appendix F

Raw values and statistical outcome of the paired sample t-tests

To ensure that the subsample that completed the survey at one-year follow-up does not differ from the whole sample at baseline, demographic variables and scores reported before and during lockdown in 2020 were compared between the two groups.

Table F1

Raw values and statistical outcome of the paired sample t-tests

	**During lockdown (Initial sample, N = 430)**	**1y post lockdown (Follow-up sample, N = 133)**	**During vs 1y post lockdown (Follow-up sample, N = 563)**
	Mean (SD)<br>Min-Max	Mean (SD)<br>Min-Max	
**FATIGUE**			
Physical fatigue	10.72 (3.76)<br>4.00-20.00	10.02 (3.53)<br>4.00-20.00	t=1.90,<br>p=.06
Mental Fatigue	11.37 (4.13)<br>4.00-20.00	10.82 (3.98)<br>4.00-20.00	t=1.36,<br>p=.18
**ANXIETY STATE**			
General anxiety	13.07 (4.23)<br>6.00-24.00	12.11 (3.80)<br>6.00-23.00	**t=2.35,<br>p=.02**
COVID-related anxiety	58.04 (29.01)<br>0-100	55.86 (30.62)<br>0-100	t=0.74,<br>p=.46
**WORK ACTIVITIES**			
Effort	61.90 (31.50)<br>0-100	60.83 (32.88)<br>1-100	t=0.34,<br>p=.74
Flexibility	72.27 (32.30)<br>0-100	74 .98 (32.75)<br>0-100	t=-0.72,<br>p=.47
Activity	58.75 (33.89)<br>0-100	60.63 (34.38)<br>0-100	t=-0.55,<br>p=.58
**LEISURE ACTIVITIES**			
Screen exposure, *min-24h*	357.87 (224.10)	377.66 (217.40)	t=-0.89,

	0-960	1-960	p=.37
Outdoor activities, *min-24h*	79.85 (84.32)<br>0-540	78.05 (79.66)<br>0-360	t=0.22,<br>p=.83
Sports, *min-24h*	37.27 (41.74)<br>0-360	39.48 (41.59)<br>0-210	t=-0.53,<br>p=.60
**MENTAL LOAD**			
House duties	63.96 (29.53)<br>0-100	68.21 (28.36)<br>0-100	t=-1.46,<br>p=.14
Social interactions	69.24 (28.63)<br>0-100	71.36 (29.37)<br>0-100	t=-0.74,<br>p=.46
At work	55.63 (31.71)<br>0-100	63.35 (26.24)<br>0-100	t=-1.78,<br>p=.08
Self-centered activities	69.01 (29.04)<br>0-100	71.52 (27.76)<br>2-100	t=-0.87,<br>p=.38
**SLEEP & SLEEPINESS**			
Sleepiness (ESS)	7.53 (4.38)<br>0-21	6.94 (4.27)<br>0-19	t=1.36,<br>p=.18
Sleep quality PSQI-C1	1.37 (0.88)<br>0-3	1.26 (0.90)<br>0-3	t=1.25,<br>p=.21
Sleep latency PSQI-C2	1.29 (1.03)<br>0-3	1.17 (0.91)<br>0-3	t=1.27,<br>p=.21
Sleep duration PSQI-C3	0.34 (0.70)<br>0-3	0.35 (0.66)<br>0-3	t=-0.09,<br>p=.93
Sleep efficiency PSQI-C4	0.55 (0.85)<br>0-3	0.53 (0.84)<br>0-3	t=0.30,<br>p=.77
Sleep disturbance PSQI-C5	1.31 (0.55)<br>0-3	1.32 (0.57)<br>0-3	t=-0.20,<br>p=.84
Sleep pills PSQI-C6	0.54 (1.04)<br>0-3	0.47 (1.00)<br>0-3	t=0.62,<br>p=.50
Daytime dysfunction PSQI-C7	1.20 (0.99)<br>0-3	1.16 (0.97)<br>0-3	t=0.39,<br>p=.70
Naps	1.17 (1.81)<br>0-7.75	1.11 (1.78)<br>0-7.25	t=0.33,<br>p=.74

s
